# Timing of menarche in Norwegian girls: associations with body mass index, waist circumference and skinfold thickness

**DOI:** 10.1186/s12887-017-0893-x

**Published:** 2017-06-06

**Authors:** Heiko Bratke, Ingvild Særvold Bruserud, Bente Brannsether, Jörg Aßmus, Robert Bjerknes, Mathieu Roelants, Pétur B. Júlíusson

**Affiliations:** 1 Department of Internal Medicine, Section of Paediatrics, Haugesund District Hospital, Haugesund, Norway; 2 Department of Paediatrics, Haukeland University Hospital, Bergen, Norway; 3 Department of Clinical Science, Section of Paediatrics, University of Bergen, 5021 Bergen, Norway; 4 Department of Paediatrics, Stavanger University Hospital, Stavanger, Norway; 5 Haukeland University Hospital, Centre for Clinical Research, Bergen, Norway; 6 KU Leuven –Department of Public Health and Primary Care, KU Leuven - University of Leuven, Leuven, Belgium

**Keywords:** Menarche age, Puberty, Overweight, BMI, Skinfold thickness, Waist circumference

## Abstract

**Background:**

Research studies show conflicting results regarding the association between menarche and body weight. The purpose of the present study was to investigate if anthropometric indicators of body composition, body mass index (BMI), waist circumference (WC), triceps (TSF) and subscapular skinfold (SSF) thicknesses, were differentially associated with age at menarche in Norwegian girls.

**Methods:**

The association between menarche and BMI, WC, TSF and SSF was investigated in 1481 girls aged 8–15.5 years, and in a subgroup of 181 girls with menarche during the 12 months prior to examination. Anthropometric measures were categorized as low (< −1SDS), average (−1 ≤ SDS ≤ +1) or high (> 1SDS), and menarche according to this classification was analysed with Kaplan-Meier curves and unadjusted and adjusted Cox regression.

**Results:**

The median age at menarche in the total sample was 13.1 years. In the unadjusted models, low categories of all traits were associated with later menarche, and high categories with earlier menarche. When adjusted for other covariates, earlier menarche was only related with a high BMI (Hazard Ratio 1.41, 95% confidence interval (CI) 1.07, 1.85), and later menarche with a low BMI (HR 0.53, 95%CI 0.38, 0.75) and low SSF (HR 0.54, 95%CI 0.39, 0.75). In girls with recent menarche, early menarche was significantly associated with a high BMI in the final model (HR 1.79, 95%CI 1.23, 2.62).

**Conclusions:**

The timing of menarche was associated with the BMI, WC, TSF and SSF, but more strongly so with the BMI. These associations may be related to a common tempo of growth, as the mean age at menarche has remained stable during the last decades during a time period while the prevalence of overweight and obesity has increased significantly.

## Background

In Western populations, the age at menarche has declined about 0.3 years per decade until the 1950’s [1, 2]. In Norwegian girls, the mean age at menarche was reported to be above 16 years in 1830, whereas it has been stable around 13 years since the 1940s [3–6]. In contrast, studies from Denmark and the US showed a further decline in the age of pubertal onset in girls, in particular the age of breast development [7, 8]. Changes in age at menarche were however modest, suggesting asymmetrical secular trends in the timing of pubertal development [7, 9].

The observed variation in the activation of the hypothalamic-pituitary-gonadal axis and menarche, reflects interactions between genetic background and various endogenous and exogenous factors [1]. Prenatal conditions, nutrition and body composition, light exposure, endocrine disrupting chemicals (EDC) and psychosocial stress have been suggested as possible regulators of this development [1, 10].

In the early 1970s, Frisch and Revelle suggested a “critical weight” theory, pointing out the relationship between weight and pubertal timing [11]. Later, the leptin hormone secreted by fat tissue, has been shown to function as a signal for energy status and as a permissive factor for pubertal onset by modulating the Kiss1/Kiss1R system [12]. The Kiss1/Kiss1R system seems to represent a link between the reproductive and the energy system, influenced not only by leptin, but also ghrelin, insulin, pro-opiomelanocortin and neuropeptide Y [13]. Therefore, one can speculate that the increase in the prevalence of overweight and obesity should result in a downward trend in pubertal timing, and several studies have provided evidence for this association [14, 15]. However, a Danish study showed that, although a relatively high BMI was consistently associated with an earlier onset of the pubertal growth spurt in children born between 1930 and 1969, such a trend was also seen in girls with a low BMI. Therefore, the BMI alone could not explain the observed downward trend [16].

In the present study, age at menarche was compared to anthropometric indicators of body composition in Norwegian girls, notably body mass index (BMI), waist circumference (WC), and triceps (TSF)- and subscapular (SSF) skinfold thickness. Our hypothesis was that measures of subcutaneous and/or central fat tissue would correlate more strongly with age at menarche compared to an overall index of body weight.

## Methods

The present analysis is based on a sample of 8–15.5 year old girls who participated in the Bergen Growth Study (BGS). This cross-sectional study on growth from birth to 19 years of age included 4035 girls measured in 2003–2006 and representative for Bergen County. All participating girls in the included primary schools (grades 1–10) were visually assessed by a study nurse for signs of breast development, and subsequently asked about menarche and, if applicable, age at menarche (month/year) [17]. From this study population, 68 girls were excluded because of diseases known to affect growth, and 4 because of incomplete information. In total, 1481 girls were between 8 and 15.5 years and thus eligible for inclusion in the present study (“total study sample”). Girls who had their menarche during the 12 months preceding the measurement represented a subgroup of “girls with recent menarche”. Age at menarche was recorded and height, weight, WC, TSF and SSF were measured according to standardized procedures [17–19]. Briefly, height was measured to the nearest 0.1 cm with a portable Holtain stadiometer (Crosswell, UK) and weight to nearest 0.1 kg with a Seca personal digital scale (Hamburg, Germany). Waist circumference was measured at the end of normal expiration with a Lufkin W606 PM metal measurement tape placed at the midpoint between the lowest rib and the top of the iliac crest. Skinfolds were measured on the left side with a Holtain Skinfold Caliper (Crosswell, UK). The TSF was measured on the back side of the upper arm midway between the acromion and the radial head. The SSF was measured 2 cm below the inferior angle of the scapula. The rationale for choosing these were threefold: [1] the triceps and subscapulae are common sites for taking skinfolds; [2] at these sites, the measurements are easier to perform and standardize compared to other measurement sites; and [3] we wanted to include both a “peripheral” (TSF) and more central or “truncal” (SSF) site. All anthropometric measures were converted to SD scores (SDS) using national growth references, and categorized as low (< −1SDS), average (−1 ≤ SDS ≤ +1) or high (> 1SDS).

### Statistical analysis

Median age at menarche was estimated with Kaplan-Meier analysis, taking into account that girls who had not yet reached menarche are right censored. The mean and variance were estimated with probit analysis of status presens data assuming a Gaussian distribution of age at menarche in the total sample, and calculated as the arithmetic mean and SD in the subgroup with recent menarche. The association between the menarche and the anthropometric measures (BMI, WC, SSF and TSF) was analysed with Cox proportional hazards models. Predictor variables were grouped as low, average or high as described above, and the average group was used as the reference category. In addition, BMI was also classified as an ordinal variable with four levels according to IOTF (International Obesity Task Force) criteria for overweight (the equivalent of a BMI ≥ 25 kg/m^2^ in adults) and obesity (equivalent of BMI ≥ 30 kg/m^2^), and the analogous criteria for underweight (the equivalent of a BMI ≤ 18.5 kg/m^2^) [20, 21]. The IOTF-classification was included because its common use in clinical and research settings. Results are presented for unadjusted simple regression models of each marker separately, fully adjusted multiple regression models including all anthropometric measures, and final models which are the result of a (backwards) stepwise removal of statistically not significant covariates (using *p* > 0.1 as a conservative criterion for removal). The sample size of the Bergen Growth Study was estimated with the aim to detect secular changes in height and weight since the 1970s. A post hoc power analysis of the present study shows that the sample allows to detect a statistically significant hazard ratio of approximately 1.3 in the analysis of all girls, and a hazard ratio of approximately 1.5 in the subgroup of girls with recent menarche.

Test results with a *p*-value less than 0.05 were considered as statistically significant. Data analysis was done using IBM SPSS release 21.0 and in R version 3.2 (R Foundation for Statistical Computing, Vienna, Austria, 2015).

## Results

Of the total study sample of 1481 girls, 477 reported menarche, of whom 181 had experienced their first menstruation during the 12 months preceding measurement (“girls with recent menarche”). In the total study sample, the Kaplan-Meier median age at menarche was 13.1 years (95%CI 13.0–13.3), while the probit mean was 13.3 years (95%CI 13.1–13.4) with a corresponding SD of 0.9 years. In girls with recent menarche, the median age at menarche was 13.2 years (95%CI 13.0–13.4), and the probit mean 13.2 (95%CI 13.0–13.3) with a corresponding SD of 0.9 years.

In the total study sample, the IOTF-defined prevalence of overweight including obesity was 14.4%, and that of underweight 9.7%, and in girls with recent menarche, 11.1% and 13.8%, respectively.

**Fig. 1:**
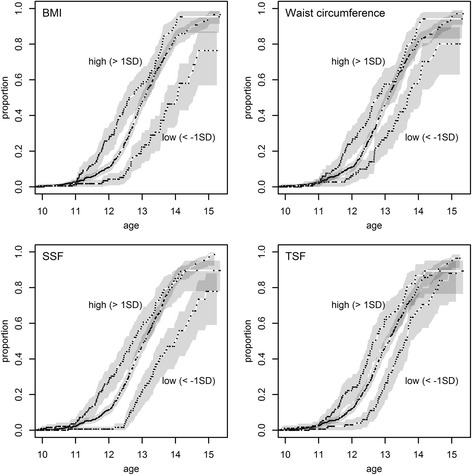
Kaplan-Meier curves of age at menarche showing that menarche occurs at an earlier age in girls with a high (> +1 SDS) BMI, Waist circumference, Subscapular (SSF) or Triceps (TSF) skinfold, and at a later age in girls with a low (< −1 SDS) value for these anthropometric indicators. The line in between shows the Kaplan-Meier estimate in girls with average (−1 to +1 SDS) measurement values. Dots are censored observations, and shaded areas indicate the 95% confidence interval

In the unadjusted analysis of the total study sample, low BMI SDS was associated with later menarche and a high BMI SDS with earlier menarche (Table 1, Fig. 1). Comparable results were obtained for WC, TSF and SSF. In the fully adjusted model, a high BMI was significantly associated with menarche at an earlier age, and both a low BMI and low SSF with menarche at a later age. Backward elimination of non-significant variables preserved the level of significance and did not alter the corresponding Hazard ratios by more than 0.04. The timing of menarche was associated with the IOTF weight classes in a similar way: median age (95%CI) was 12.5 (12.1–13.0) years in girls with overweight including obesity (*n* = 213), 13.1 (13.0–13.2) years in girls with normal weight (*n* = 1124), and 14.1 (13.7–14.6) years in girls with underweight (*n* = 144).

**Table 1:** Kaplan-Meier estimates and Cox regression of menarche according to the BMI, WC, TSF and SSF^a^ in the total sample and in girls with recent menarche

			Unadjusted model^b^		Fully adjusted model^b^		Final model^b^	
	*N*	Median	HR (95% CI)	*P*	HR (95% CI)	*P*	HR (95% CI)	*P*
All girls (*N* = 1481)								
BMI								
Low	262	14.1	0.40 (0.30–0.55)	<0.001	0.56 (0.39–0.82)	0.001	0.53 (0.38–0.75)	<0.01
Normal	960	13.1	1.00		1.00		1.00	
High	259	12.6	1.46 (1.16–1.84)	0.001	1.45 (1.03–2.05)	0.01	1.41 (1.07–1.85)	0.01
WC								
Low	236	13.7	0.54 (0.40–0.71)	<0.001	0.93 (0.67–1.30)			
Normal	1000	13.1	1.00		1.00			
High	244	12.8	1.33 (1.04–1.71)	0.01	0.86 (0.59–1.24)			
TSF								
Low	235	13.6	0.59 (0.45–0.78)	<0.001	0.97 (0.72–1.30)			
Normal	988	13.1	1.00		1.00			
High	248	12.7	1.33 (1.04–1.71)	0.01	1.01 (0.73–1.41)			
SSF								
Low	237	14	0.40 (0.29–0.54)	<0.001	0.53 (0.37–0.75)	<0.001	0.54 (0.39–0.75)	<0.01
Normal	965	13.1	1.00		1.00		1.00	
High	273	12.8	1.21 (0.95–1.54)		1.03 (0.73–1.45)		0.96 (0.72–1.27)	
Girls with recent menarche (*N* = 181)								
BMI								
Low	26	13.60	0.76 (0.50–1.17)		0.92 (0.51–1.66)		0.76 (0.50–1.17)	
Normal	118	13.10	1.00		1.00		1.00	
High	37	12.70	1.79 (1.23–2.62)	0.001	1.19 (0.67–2.12)		1.79 (1.23–2.62)	0.003
WC								
Low	19	13.70	0.82 (0.50–1.33)	<0.001	0.99 (0.52–1.87)			
Normal	131	13.20	1.00		1.00			
High	31	12.60	1.96 (1.32–2.94)	0.01	1.40 (0.79–2.48)			
TSF								
Low	36	13.50	0.80 (0.55–1.17)		1.08 (0.69–1.70)			
Normal	119	13.10	1.00		1.00			
High	23	12.70	1.74 (1.11–2.74)		1.11 (0.61–2.04)			
SSF								
Low	25	13.50	0.65 (0.42–1.00)	0.050	0.66 (0.39–1.14)			
Normal	124	13.10	1.00		1.00			
High	32	12.70	1.71 (1.15–2.53)	0.01	1.25 (0.73–2.13)			

^a^Grouped as low (< −1 SDS), normal (> −1 SDS – > +1 SDS) or high (> +1 SDS) BMI, WC, TSF and SSF. Estimates and models based on the total sample take into account that some girls have not yet reached menarche (censored)

^b^ Unadjusted models are simple Cox regression models for each marker separately; fully adjusted models are multiple Cox regression models including all anthropometric markers; and final models are multiple Cox regression models after backward elimination of statistically not significant covariates (*p* > 0.1)

The same trends were observed in the group of girls with recent menarche, but with a lower significance level, probably because of the smaller sample size (Table 1). Also, the effect sizes were usually larger for the high categories of BMI, WC, TSF and SSF, and smaller for the low categories, when compared to the total cohort.

## Discussion

In the present study, we found a median age at menarche in Norwegian girls of 13.1 years. Although the timing of menarche was strongly related to all weight related anthropometric variables, early menarche was only significantly associated with a high BMI, and late menarche with a low SSF or low BMI in multiple Cox regression analysis.

The mean age of menarche in Norwegian girls has been stable for more than half a century, while the prevalence of overweight and obesity has continued to rise during the past decades [22, 23]. Rosenberg et al. reported the age at menarche between 1830 and 1960 using recall data from Norwegian maternity hospitals, and showed a steady decline that reached a nadir just above 13 years of age in the 1940s [3]. More recently, comparable estimates of age at menarche in Norwegian girls have been published by Tell et al. [5], using youth data from Oslo, and by Bratberg et al., using data collected from 1995 to 2001 for the Young-HUNT study [6]. The estimates from our own study conclude this list and confirm that age at menarche in Norwegian girls has not changed since the 1940s. The current median age at menarche of 13.1 years in Norwegian girls is similar to that observed in other North-European countries [1]. Recent findings from the USA, Denmark and the Netherlands showed a slight decrease in menarcheal age during the last decade [7, 9, 24]. We could not document such a trend, in Norwegian girls.

All weight-related anthropometric variables were strongly associated with the timing of menarche. A relatively low value of the BMI, WC, TSF and SSF was associated with menarche at a later age, and a relatively high value with an earlier age at menarche. Earlier studies have linked early menarche to higher weight status in childhood [25, 26], and Adair et al. found a higher prevalence of overweight in girls with menarche before 11 years compared to those with a menarche after 14 years of age [27]. This difference remains up to young adulthood at least, as follow-up of young women with early menarche showed higher BMI and larger skinfold-thickness at the age of 21 and 27 years, when compared to women with late menarche [28]. Finally, using data from 34 European countries and North America, Currie et al. could show that at on an individual level, age at menarche was 1 month earlier for each unit increase in BMI [14]. Further, mean age at menarche was approximately 1 week earlier with each percentage point increase in prevalence of overweight/obesity at country level [14]. About 14.4% of the girls in our study were overweight according to the IOTF. Although this estimate roughly corresponds to a threefold increase in the prevalence of overweight during the past decades [22], it is surprising that the age at menarche has not changed during this period. This indicates that the mechanism behind the association between weight status and tempo of maturation, at least for menarche as an endpoint, may be more complex than a direct causal relation.

In the multiple Cox regression model, a relatively high BMI was related with early menarche and low BMI or SSF was with late menarche. Our hypothesis was that measures of subcutaneous fat tissue like TSF and SSF, would show a stronger relation with menarche than the BMI, which measures both fat mass and lean mass. However, the present findings show the opposite, as we found the BMI to be a consistent predictor of early and late menarche. This suggests a strong association between the increase in BMI and tempo of maturation, and could explain why menarche is more closely related to the BMI than to measures of subcutaneous (skinfolds) or central (WC) fat tissue. However, the later menarche in girls with a low SSF is equally interesting as one can speculate that SSF, as a measure of truncal subcutaneous fat tissue, has more impact on maturational processes than TSF, which is a measure of peripheral fat tissue.

A limitation in the present work is that girls might have changed weight status between menarche and the time of examination, in particular during puberty. Because of this possibility, we repeated our analysis in a subsample of girls who had their menarche within 12 months (mean time since menarche was 6.3 months) prior to the date of measurement [29]. The analysis of this subgroup showed the same trends as in the total study group, although not always statistically significant, probably due to the loss of statistical power because of the much smaller sample size. A further limitation is the cross-sectional nature of the data. Because of this, no causal conclusions can be made.

## Conclusion

Although timing of menarche was clearly associated with weight-related anthropometric measures in Norwegian girls, the increasing prevalence of overweight and obesity seems to have had little impact on the mean age at menarche. Therefore, the observed association between weight-related anthropometric measures and menarche might be reflecting maturation to a larger extent than the degree of adiposity. This is also supported by the findings in the current study that anthropometric measures of subcutaneous fat tissue (TSF, SSF) and central fat (WC) did not show stronger relationship to menarche than BMI.

## Abbreviations

BMI: Body mass index
EDC: Endocrine disrupting chemicals
IOTF: International obesity task force
SSF: Subscapularis skinfolds
TSF: Triceps skinfolds
WC: Waist circumference
